# MaasPenn Radiomics Reproducibility Score: A Novel Quantitative Measure for Evaluating the Reproducibility of CT-Based Handcrafted Radiomic Features

**DOI:** 10.3390/cancers14071599

**Published:** 2022-03-22

**Authors:** Abdalla Ibrahim, Bruno Barufaldi, Turkey Refaee, Telmo M. Silva Filho, Raymond J. Acciavatti, Zohaib Salahuddin, Roland Hustinx, Felix M. Mottaghy, Andrew D. A. Maidment, Philippe Lambin

**Affiliations:** 1 The D-Lab, Department of Precision Medicine, GROW-School for Oncology, Maastricht University, 6229 ER Maastricht, The Netherlands; t.refaee@maastrichtuniversity.nl (T.R.); z.salahuddin@maastrichtuniversity.nl (Z.S.); philippe.lambin@maastrichtuniversity.nl (P.L.); 2 Department of Radiology and Nuclear Medicine, Maastricht University Medical Center+, 6229 HX Maastricht, The Netherlands; felix.mottaghy@mumc.nl; 3 Division of Nuclear Medicine and Oncological Imaging, Department of Medical Physics, CHU de Liege, CRC In Vivo Imaging, University of Liège, 4000 Liege, Belgium; rhustinx@chu.ulg.ac.be; 4 Department of Nuclear Medicine and Comprehensive Diagnostic Center Aachen (CDCA), University Hospital RWTH Aachen University, 52074 Aachen, Germany; 5 Department of Radiology, Perelman School of Medicine, University of Pennsylvania, Philadelphia, PA 19104, USA; bruno.barufaldi@pennmedicine.upenn.edu (B.B.); racci@pennmedicine.upenn.edu (R.J.A.); andrew.maidment@pennmedicine.upenn.edu (A.D.A.M.); 6 Department of Diagnostic Radiology, Faculty of Applied Medical Sciences, Jazan University, Jazan 45142, Saudi Arabia; 7 Department of Statistics, Federal University of Paraíba, João Pessoa 58051-900, Brazil; tmfilho@gmail.com

**Keywords:** radiomics reproducibility, harmonization, ComBat

## Abstract

**Simple Summary:**

The reproducibility of handcrafted radiomic features (HRFs) has been reported to be affected by variations in imaging acquisition and reconstruction parameters. However, to date, these effects have not been understood or quantified. In this study, we analyzed a significantly large number of scenarios in an effort to quantify the effects of variations on the reproducibility of HRFs. In addition, we assessed the performance of ComBat harmonization in each of the 31,375 investigated scenarios. We developed a novel score that can be considered the first attempt to objectively assess the number of reproducible HRFs in different scenario. Following further validation, the score could be used to decide on the inclusion of data acquired differently, as well as the assessment of the generalizability of developed radiomic signatures.

**Abstract:**

The reproducibility of handcrafted radiomic features (HRFs) has been reported to be affected by variations in imaging parameters, which significantly affect the generalizability of developed signatures and translation to clinical practice. However, the collective effect of the variations in imaging parameters on the reproducibility of HRFs remains unclear, with no objective measure to assess it in the absence of reproducibility analysis. We assessed these effects of variations in a large number of scenarios and developed the first quantitative score to assess the reproducibility of CT-based HRFs without the need for phantom or reproducibility studies. We further assessed the potential of image resampling and ComBat harmonization for removing these effects. Our findings suggest a need for radiomics-specific harmonization methods. Our developed score should be considered as a first attempt to introduce comprehensive metrics to quantify the reproducibility of CT-based handcrafted radiomic features. More research is warranted to demonstrate its validity in clinical contexts and to further improve it, possibly by the incorporation of more realistic situations, which better reflect real patients’ situations.

## 1. Introduction

In recent decades, we have witnessed a leap in the development of medical imaging and computational power. Combined with advances in artificial intelligence (AI), the opportunity for converting medical images into mineable quantitative data was created and the field of radiomics emerged as a result [1]. Radiomics—the high throughput extraction of mineable quantitative features from medical imaging [2]—gained exponential research momentum within the last decade based on a series of handcrafted feature measures that have been developed. The future of radiomics is rife with opportunities from providing alternatives for invasive diagnostic procedures, to playing a significant role in early disease detection and personalized treatment management [3]. Due to the heterogeneity of tumors [4,5], clinical approaches such as tissue biopsies, never characterize the entirety of the tumor and frequently require repeated tissue sampling [6]. In contrast, radiomics can assess entire regions of interest (ROIs), providing better characterization of the lesion [7]. Moreover, radiomics is fast, non-invasive, highly accurate, and supplies potential cost-effective clinical biomarkers, which will ultimately improve personalized care.

Handcrafted radiomic features (HRFs) can extract biological information from the tissue under study [3] for use as potential clinical biomarkers. To date, many studies have reported on the potential of HRFs to predict clinical endpoints, such as detection and diagnosis, response to treatment, overall survival, and progression free survival [8,9,10]. However, a number of limitations hinder the clinical translation of radiomics. Quantitative biomarkers must be reproducible and robust [11]. As HRFs are calculated using data characterization algorithms applied to the medical image, changes in scan acquisition and reconstruction can significantly affect HRFs. A substantial fraction of HRFs has been reported to be sensitive to variations in the acquisition and reconstruction parameters of the scans, and the number of reproducible HRFs is usually dependent on the degree of variation in these parameters [12,13,14,15,16].

Multiple studies have investigated the potential of feature harmonization methods, such as ComBat, to mitigate variations attributable to differences in acquisition and reconstruction parameters [17,18,19,20]. ComBat harmonization was originally developed to harmonize gene expression arrays [21] and has shown promising results in radiomics analyses in certain scenarios [17,18,19,20]. However, there is no consensus on how or when to use ComBat harmonization in radiomics.

We previously published a framework to assess the reproducibility of radiomic features [7], with two follow up studies to validate it on a phantom dataset [12,14]. A number of studies investigated the effects of different parameters individually on the reproducibility of HRFs [22,23]. However, the collective effect of variations in more than a single imaging parameter at a time is yet to be investigated. As the above mentioned studies highlighted the effects of variations in imaging parameters, a reproducibility analysis is required to ensure the development of robust signatures. However, since the majority of radiomics studies included datasets collected retrospectively, the planning and execution of reproducibility analysis is a long and extensive process. Therefore, there is a strong need for an objective quantitative measure that can assess the concordance in HRFs’ values across scans acquired differently.

In this study, we investigated the effect of variations in imaging parameters on different imaging scenarios using computed tomography (CT) scans of phantoms. Our aim was to develop an objective metric to assess the reproducibility of HRFs across scans, which could be used as an indicator to assess the agreement in HRFs values extracted from the scans under analysis, and further to act as a tool to ‘quality check’ radiomic studies.

## 2. Materials and Methods

### 2.1. Imaging Data

**Figure 1:**
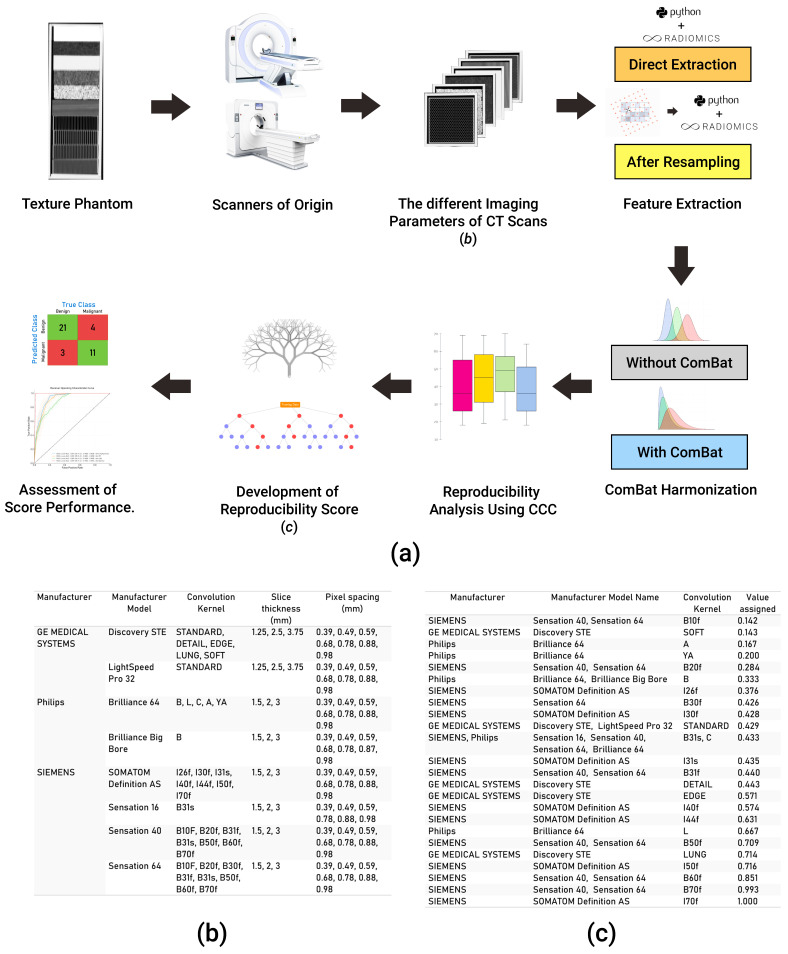
Explanatory diagram of our workflow. (**a**) The steps undertaken for data collection, analysis, and the development of the score; (**b**) a table showing different acquisition and reconstruction parameters of the phantom dataset; (**c**) a table showing the numeric value assigned to each kernel in the data analyzed.

The publicly available Credence Cartridge Radiomics phantom dataset [24] was analyzed in this study (available on: TCIA.org, accessed on 6 July 2020) [25]. The dataset consists of 251 scans of a phantom that were acquired with different imaging vendors, models, and imaging parameters (Figure 1b). The workflow applied in this study is shown in Figure 1a.

### 2.2. Volumes of Interest and HRF Extraction

Each layer of the phantom (in total, 10 layers) was subdivided into 16 equal volumes of interest (VOI), sized 2 × 2 × 2 cm^3^. A total of 160 VOIs were segmented per scan, resulting in a total of 40,160 VOIs. HRFs were extracted using the open source PyRadiomics software version 2.2.0 [26]. HRFs were extracted three different times: directly from the original scans; following resampling of all scans to the median resolution available in the dataset; and following resampling of all scans to the lowest resolution available in the dataset. Image intensities were binned in all of the three scenarios with a binwidth of 25 Hounsfield Units (HUs) to reduce noise levels and texture matrix sizes, and therewith the required computational power. No further image preprocessing was applied in any of the scenarios. Extracted HRFs included HU intensity features, and texture features that describe the spatial distribution of voxel intensities using five matrices: gray-level co-occurrence (GLCM); gray-level run-length (GLRLM); gray-level size-zone (GLSZM); gray-level dependence (GLDM); and neighborhood gray-tone difference (NGTDM) matrices. A more detailed description of PyRadiomics HRFs can be found online at https://pyradiomics.readthedocs.io/en/latest/features.html (accessed on 7 January 2021).

### 2.3. Exploratory Analysis

All statistical analyses were performed using R [27] (RStudio, V 3.6.3) [28]. We performed an initial exploratory analysis to assess the reproducibility of HRFs in the different scenarios mentioned above, as well as the use of ComBat harmonization [21] and Cosine Windowed Sinc (CWS) image interpolation [29]. The concordance correlation coefficient (CCC) was used to assess the reproducibility of HRFs across the different pairwise scenarios [30], using epiR (V 2.0.26) [31]. The CCC measures the concordance in both value and rank in each of the pairwise scenarios. HRFs with CCC > 0.9 were considered reproducible. The reproducibility of HRFs was assessed in: (i) HRFs extracted from the original scans, before and after ComBat harmonization; (ii) HRFs extracted from scans resampled to the median voxel size (0.68 × 0.68 × 1.5 mm^3^), before and after ComBat harmonization; and (iii) HRFs extracted from scans resampled to the largest voxel size (0.98 × 0.98 × 3.75 mm^3^), before and after ComBat harmonization.

### 2.4. Evaluation of the Effects of Variations in Imaging Parameters

To unravel the effects of variations in imaging parameters, we assessed the reproducibility of HRFs across each pair of the 251 scans, resulting in a total of 31,375 pairs (scenarios) analyzed. Each of the eight parameters: vendor; model; tube current; exposure; exposure time; slice thickness; pixel spacing; and convolution kernel was given a numeric value between 0 and 1 depending on the scenario. For vendor and model, we assigned a binary value of 0 if the vendor/model is different across the pairs, and 1 if the same vendor/model was used to acquire both scans in the scenario. For the remaining parameters, a value between 0 and 1 was calculated by dividing the minimum value of a given parameter by the maximum value across the pairs being analyzed. Convolution kernels were assigned a numeric value based on a schema, which ranked the limiting frequency of the kernel (Figure 1c). To assess the impact, as well as the predictive power, of the variations in imaging parameters on the percentage of reproducible HRFs in different scenarios, a random forest model [32] was applied.

### 2.5. Quantitative Score Development

We trained a regression random forest model on the 31,375 pairs, using the eight parameters as predictors: vendor; model; tube current; exposure; exposure time; slice thickness; pixel spacing; and convolution kernel, while the number of reproducible HRFs per scenario was used as the outcome. The parameters with the largest feature importance in the model were used to develop a quantitative score. The default parameters for the random forest on RandomForest package (V 4.6) were used (including number of trees: 500), except for the number of variables per split, which was set to three. The most important parameters contributing to the model were multiplied by their importance and divided by the total importance of the included parameters. The sum of weighted parameters was used as a quantitative score with values ranging between ~0.3 and 1. The correlation of the developed score with the percentage of reproducible HRFs across the investigated scenarios was assessed using spearman correlation [33].

To develop a methodology for applying the developed score in radiomic studies, we used different thresholds (increments of 10% between 10% and 90%) of the percentage of reproducible features across the scenarios. The thresholds were used to create a binary label for the percentage of reproducible HRFs in a given scenario, where 0 indicated that the number of reproducible HRFs was below the threshold, and 1 indicated that the percentage was higher than the threshold. Receiver operating curve (ROC) analyses were performed using the binary status of pairs as the outcome to select the best threshold of the calculated score for classifying the scans as above and below a certain threshold. The performance of the cut-off point score was assessed for each of the thresholds defined.

To assess the robustness of the quantitative score, the analysis was repeated 100 times, and the scenarios (pairs) were split randomly into 80% training and 20% validation in each of the runs. Area under the receiver operator characteristics curve (AUC) [34], sensitivity and specificity [35] were used to assess the performance of the developed score in predicting whether the percentage of reproducible HRFs in a given scenario was above the selected threshold.

To identify HRFs that were insensitive to variations in imaging parameters, the intersection of reproducible HRFs across all the scenarios was obtained. Similarly, HRFs that were harmonizable using ComBat harmonization [21] and/or CWS interpolation were identified by obtaining the intersection of HRFs that were found to be reproducible across all pairs following the application of a given harmonization method.

## 3. Results

### 3.1. Extracted HRFs

A total of 91 original HRFs were extracted with PyRadiomics toolbox. These HRFs were divided into: 18 first order statistics, 22 GLCM, 14 GLDM, 16 GLRLM, 16 GLSZM, and 5 NGTDM HRFs.

### 3.2. The Reproducibility of HRFs across Pairs

The number (percentage) of reproducible HRFs extracted directly from the original images varied depending on the differences in imaging parameters across each of the analyzed pairs, with a mean of 25.6 (28.1%) HRFs and a standard deviation of 14.4. The average numbers of reproducible HRFs following image resampling to the median and lowest resolutions were 29 (31.9%) +/− 16.6, and 26 (28.6%) +/− 15.5.

### 3.3. Reproducible and Harmonizable HRFs

**Figure 2:**
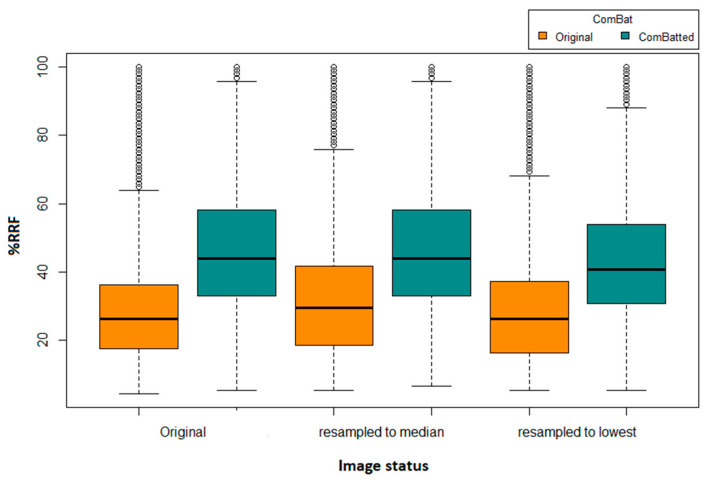
Boxplot of the number of reproducible HRFS (RRFs) across the different scenarios.

We identified four HRFs that were insensitive to all variations in the investigated 31,375 scenarios. These HRFs are: (i) original first order mean; (ii) original first order median; (iii) original first order root mean squared; and (iv) original first order total energy. One additional HRF (original first order energy) was found to be reproducible across all scenarios following image resampling both to the median and to the largest voxel size available using CWS interpolation. Similarly, one additional HRF was found to be reproducible across all scenarios following the application of ComBat harmonization on HRFs extracted from original scans (original first order 10 percentile), or from scans after resampling to the largest voxel size available (original first order energy). Two additional HRFs (original first order 10 percentile and original first order energy) were found to be reproducible across all pairs following the application of ComBat harmonization on HRFs extracted following resampling to the median voxel size available. The reproducibility and harmonizability (using ComBat or image resampling) of the remaining HRFs were dependent on the variations in imaging parameters across the pairs being analyzed. On average, ComBat harmonization outperformed image resampling. The distributions of the percentages of reproducible features in all of the investigated scenarios are shown in Figure 2.

### 3.4. The Effects of Variations in Imaging Parameters

The convolution kernel was found to be the most important factor affecting the reproducibility of HRFs. The second most important factor was found to be the slice thickness, followed by pixel spacing. The initial random forest was able to explain 81.3% of the variance in the percentage of reproducible HRFs in all of the scenarios investigated.

### 3.5. Maastricht-Pennsylvania Radiomics Reproducibility Score (MassPenn Score)

**Figure 3:**
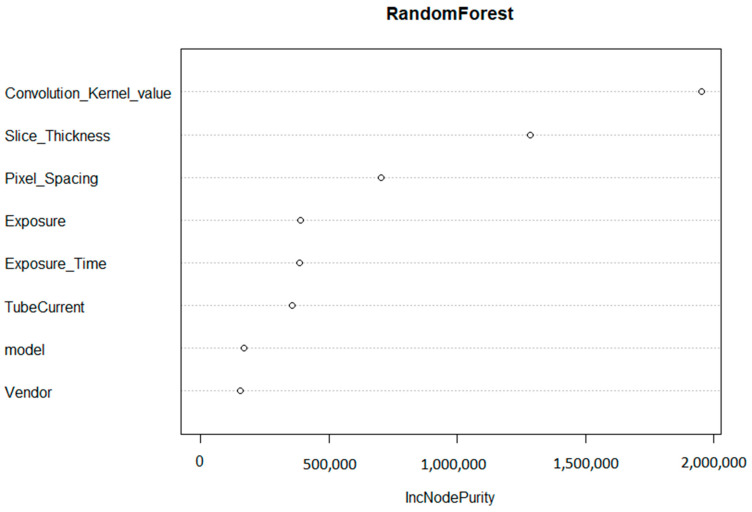
Variable importance of the regression random forest model.

Based on the importance of the variables in the random forest model (Figure 3), the convolution kernel had the highest contribution to the score with 48% of the total score. The slice thickness and pixel spacing corresponded to 33% and 19%, respectively. If the scans were acquired with the same (or similar) convolution kernel, the same slice thickness, and pixel spacing (MaasPenn score > 0.98), then the probability of having 90% or more of the HRFs reproducible is 0.97, with a 3% false alarm rate. In contrast, the probability of having 10% or less reproducible HRFs across scans acquired with different convolution kernels and voxel sizes (MaasPenn score < 0.75) is 0.74, and a 19% false alarm rate. The predictive power of our developed score to determine the percentages (thresholds) of reproducible HRFs across scans acquired differently is reported in Table 1.

### 3.6. Robustness of MaasPenn Radiomics Reproducibility Score

**Figure 4:**
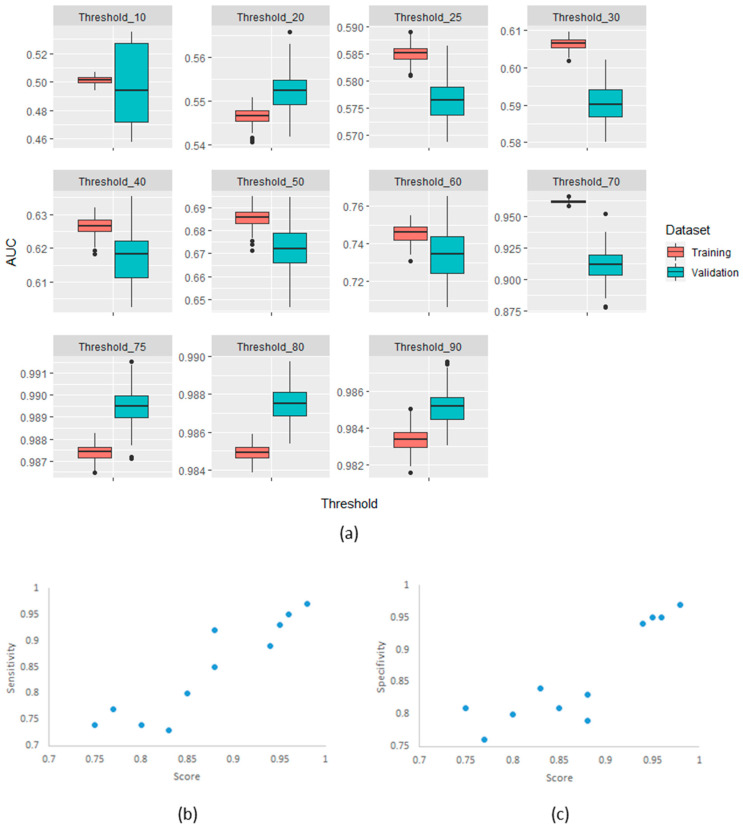
(**a**) AUC distributions across 100-runs for MaasPenn radiomics reproducibility score in the training and validation datasets for each of the thresholds of percentage reproducible HRFs; (**b**) The sensitivity as a function of the score and threshold; and (**c**) The specificity as a function of the score and threshold.

The confirmatory analysis of the robustness of the MaasPenn radiomics score was based on an experiment with 100 runs. The results showed a narrow distribution of values across the different metrics with similar performance in the training and validation sets. Figure 4 shows the distributions of AUC values on the training and validation datasets across the 100 runs.

## 4. Discussion

In this study, we investigated the effects of variations in CT imaging parameters on the reproducibility of HRFs using a phantom dataset. The scans (*n* = 251) were acquired using a wide range of imaging parameters on different imaging vendors and models. The imaging parameters can be classified into three groups: (i) resolution parameters: convolution kernel, slice thickness and pixel spacing; (ii) noise parameters: mAs, exposure and exposure time; and (iii) hardware make: vendor and model. Our analysis showed that variations in resolution parameters had the most pronounced effect on the reproducibility of HRFs, with the convolution kernel being the most significant contributor, which is concordant with previous studies [16,36,37]. Scans acquired with the same or similar convolution kernel showed the highest numbers of reproducible HRFs across scenarios. Slice thickness and pixel spacing were the other major contributors to the reproducibility of CT-based HRFs. An important finding in this study is that differences in imaging vendor and model did not seem to affect the reproducibility of HRFs significantly, given that the remaining parameters were similar/homogenous.

We further identified the HRFs that were reproducible regardless of the variations in imaging parameters in our dataset. These were strictly first order features that are descriptive of the HUs in the defined VOIs. This finding can be supported by the fact that HUs are normalized to air and water, and the subject of routine quality assurance. Hence, HRFs such as mean or median HU value are expected to be reproducible across all imaging variations. Lu et al. investigated the reproducibility of HRFs by reconstructing raw CT scans of 32 lung cancer patients using different imaging parameters, which ultimately resulted in 15 different scenarios [15]. The authors reported that 23/89 (25.8%) HRFs were found to be reproducible across their investigated scenarios, which is also in concordance with our finding that, on average, ~26/91 (28.1%) of the HRFs were found to be reproducible across all investigated scenarios. In addition, we identified HRFs that can be harmonized with ComBat or CWS image resampling regardless of the variations in imaging parameters across the scans being analyzed. Both methods could harmonize 1% additional HRFs, and the combination of ComBat harmonization and resampling to median voxel size resulted in an additional 2% of the HRFs across all scenarios. The ability of both methods to harmonize the remaining HRFs was dependent on the variations in imaging parameters in the scenarios analyzed. These findings are in line with our previous experiments, which also showed that the reproducibility and harmonizability of the majority of HRFs are dependent on the variations in imaging parameters [12,13,14,38]. In addition, they add to the body of evidence showing the need for resolution-insensitive HRFs, and/or HRFs-specific harmonization methods.

**Figure 5:**
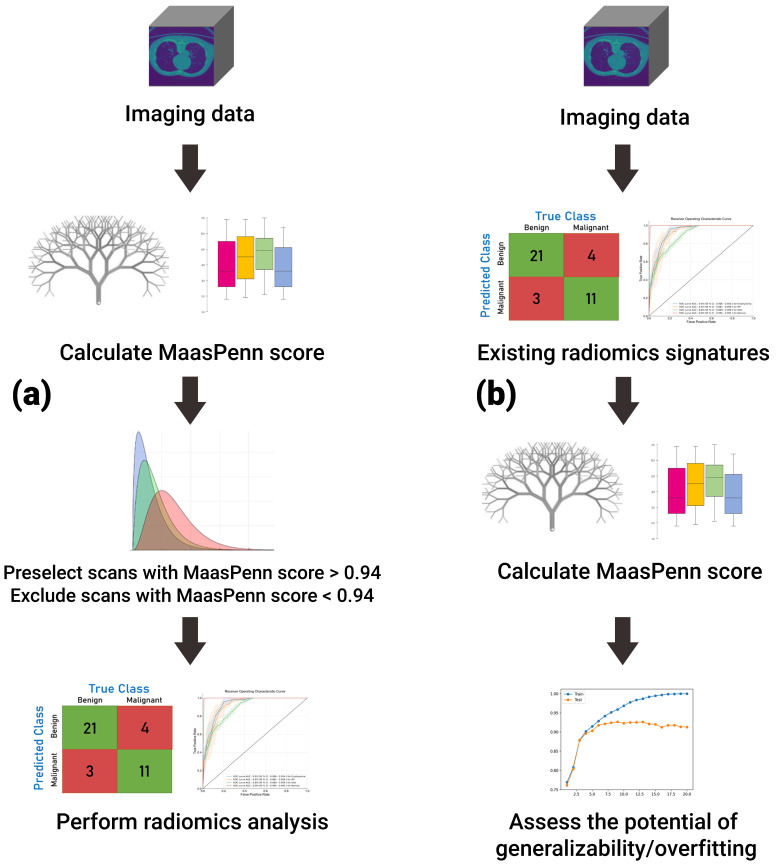
The proposed workflow of MaasPenn score for (**a**) planning new analyses; and (**b**) evaluating previously developed signatures.

Importantly, we have used the feature analysis to develop a quantitative score (MaasPenn radiomics reproducibility score), which can estimate the percentages of reproducible HRFs across CT scans acquired differently. The MaasPenn radiomics score is the first quantitative tool for assessing the reproducibility of CT-based HRFs. It can also serve as a screening tool for the inclusion of CT scans in a dataset/study (Figure 5). In prospective use (Figure 5a), following the collection of imaging datasets, the MaasPenn score can be calculated for all the scans, and only scans with a MaasPenn score higher than 0.94 (70% or more of HRFs are reproducible) should be included in further analyses. For retrospective use (Figure 5b), the MaasPenn score can be calculated on the dataset(s) used to create the signature. If the majority of scan pairs has MaasPenn higher than 0.94, then there is a high probability that the signature is generalizable. We performed additional analyses to assess the robustness of our developed score. The results showed very narrow distributions of performance metric values that were consistent on the training and validation sets across the 100 random splits, which suggests that MaasPenn radiomics reproducibility score is robust.

While the phantom dataset analyzed included a large number of scans acquired with a wide variety of imaging vendors and parameters, a number of the CT imaging vendors used in some clinics were not available for analysis in this study. As such, and despite the large number of scenarios investigated, the generalizability of MaasPenn radiomics score to CT scans acquired with those imaging vendors/parameters is yet to be investigated. In addition, we have only investigated the possibility of developing a quantitative score on a single bindwith that is most commonly used in CT radiomics. Furthermore, while the phantom used in this study was designed specifically for radiomics, it might not reflect the exact situation of real patients. Nevertheless, previous studies reported large similarities between phantom and patient studies [22,39,40]. The applicability of this score is expected to be limited to non-contrast enhanced CT radiomics, unless further validation shows otherwise. Future studies that include cadaveric/3D-printed tissues scanned with a larger number of imaging vendors/parameters can better represent patient scans, and could further enhance the utility of the MaasPenn radiomics score.

## 5. Conclusions

In conclusion, we developed the MaasPenn score, which can be considered a first at-tempt to introduce a quantitative metrics to quantify the reproducibility of CT-based handcrafted radiomic features. Following further validation, the score could be used for planning new analyses, as well as evaluating the generalizability of developed radiomic signatures. Furthermore, there is a significant need for the development of HRFs-specific harmonization methods. Further research with a larger number of scans of cadaveric/3D-printed tissues can further improve the predictive power of the MaasPenn radiomics score. The development of HRFs that are insensitive to variations in imaging parameters is another potential solution for developing generalizable radiomic signatures.

## Figures and Tables

**Table 1:** Performance of the score threshold for the identification of different HRFs reproducibility thresholds.

Percentage RRFs	Score	AUC	CI 95% Lower	CI 95% Upper	Specificity	Sensitivity	False Alarm
Threshold 10%	0.75	0.86	0.855	0.867	0.81	0.74	0.19
Threshold 20%	0.77	0.85	0.842	0.851	0.76	0.77	0.24
Threshold 25%	0.80	0.85	0.843	0.852	0.80	0.74	0.20
Threshold 30%	0.83	0.86	0.851	0.86	0.84	0.73	0.16
Threshold 40%	0.85	0.87	0.868	0.878	0.81	0.80	0.19
Threshold 50%	0.88	0.90	0.892	0.904	0.83	0.85	0.17
Threshold 60%	0.88	0.92	0.91	0.925	0.79	0.92	0.21
Threshold 70%	0.94	0.96	0.952	0.966	0.94	0.89	0.06
Threshold 75%	0.95	0.97	0.967	0.977	0.95	0.93	0.05
Threshold 80%	0.96	0.98	0.971	0.983	0.95	0.95	0.05
Threshold 90%	0.98	0.99	0.982	0.996	0.97	0.97	0.03

## Data Availability

Data is publicly available on: (https://wiki.cancerimagingarchive.net/pages/viewpage.action?pageId=39879218, accessed on 6 July 2020).
